# Malocclusion, psycho-social impacts and treatment need: A cross-sectional study of Tanzanian primary school-children

**DOI:** 10.1186/1472-6831-8-14

**Published:** 2008-05-06

**Authors:** Matilda Mtaya, Anne N Astrom, Pongsri Brudvik

**Affiliations:** 1 Department of Odontology-Community Dentistry, UoB, Norway; 2 Centre for international health, UoB, Norway; 3 Muhimbili University of Health and Allied Sciences, Dar es Salaam, Tanzania; 4 Department of Odontology- Orthodontics, UoB, Norway

## Abstract

**Background:**

studies on the relationship between children's malocclusion and its psycho-social impacts are so far largely unexplored in low-income countries. This study aimed to assess the prevalence of malocclusion, reported dental problems and dissatisfaction with dental appearance among primary school children in Tanzania. The relationship of dissatisfaction with socio-demographic characteristics, clinically defined malocclusion and psychosocial impacts of dental anomalies was investigated. Orthodontic treatment need was estimated using an integrated socio-dental approach.

**Method:**

One thousand six hundred and one children (mean age 13 yr) attending primary schools in the districts of Kinondoni and Temeke completed face to face interviews and a full mouth clinical examination. The survey instrument was designed to measure a Kiswahili translated and culturally adapted Child Oral Impact on Daily Performance (Child-OIDP) frequency score, reported dental problems, dissatisfaction with dental appearance/function and socio-demographic characteristics.

**Results:**

The prevalence of malocclusion varied from 0.9% (deep bite) to 22.5% (midline shift) with a total of 63.8% having at least one type of anomaly. Moderate proportions of children admitted dental problems; ranging from 7% (space position) to 20% (pain). The odds ratio of having problems with teeth position, spaces, pain and swallowing if having any malocclusion were, respectively 6.7, 3.9, 1.4 and 6.8. A total of 23.3% children were dissatisfied with dental appearance/function. Children dissatisfied with their dental appearance were less likely to be Temeke residents (OR = 0.5) and having parents of higher education (OR = 0.6) and more likely to reporting problem with teeth position (OR = 4.3) and having oral impacts (OR = 2.7). The socio-dental treatment need of 12% was five times lower than the normative need assessment of 63.8%.

**Conclusion:**

Compared to the high prevalence of malocclusion, psycho social impacts and dissatisfaction with appearance/function was not frequent among Tanzanian schoolchildren. Subjects with malocclusion reported problems most frequently and malocclusion together with other psycho-social impact scores determined children's satisfaction with teeth appearance- and function.

## Background

It is generally accepted that the main benefit of orthodontic treatment relates to improvements in oral function and oro-facial aesthetics and thus to improved oral health related quality of life [1-3]. A recent review on the impact of malocclusion on quality of life based on studies from industrialized countries concluded that patients are motivated to seek orthodontic care due to the physical, psychological and social effects of malocclusion [3,4]. Thus, information regarding the psycho social impacts of malocclusion is important in providing understanding of the demand for orthodontic treatment beyond clinical indicators [3,4]. Valid and reliable oral health related quality of life instruments for use among children are emerging and have the potential to provide information about the subjectively experienced consequences of oral diseases including malocclusion, the effect of malocclusion if left untreated and to facilitate appropriate treatment need assessment for dental service planning [3,5-8]. However, values attributed to dental esthetics and functioning vary according to social and cultural contexts and studies regarding the relationship between malocclusion and its psycho social impacts is so far largely unexplored in low income countries [9-14]. Recent studies of Nigerian adolescents suggest that consciousness of malocclusion does not agree with the objectively determined orthodontic treatment need [15-17]. In Tanzania, studies investigating the functional and behavioral consequences of malocclusion in children are either non-existent or very few [9,18]. This is noteworthy as normatively assessed orthodontic treatment needs based on clinical indicators alone are commonly found to vary according to age, to be high (60–90%) and thus are unlikely to be met due to the high costs of treatment that goes beyond the financial capabilities of this country [19,20]. Three quarters of the low-income countries lack sufficient human and financial resources to provide an essential health care package for their children [21].

Considering the impracticality and inappropriateness of a normative approach to the assessment of children's need for orthodontic treatment, Gherunpong et al [11] developed a new theoretical framework and model for estimating orthodontic treatment need in children. In their model they integrated clinical measures of orthodontic anomalies with children's feeling of impacts related to appearance and function as well as with measures of their oral health related behaviors. This socio-dental system for need assessment includes three levels. The first level refers to standard normative need assessment and is based solely on professionally judged malocclusions that normally require orthodontic treatments. The second level refers to impact related need assessment and relies on the integration of normative need with OHRQoL. Children who have both normative needs and their oral quality of life impaired by malocclusion are considered to have impact related need for orthodontic treatment. Propensity related need assessment (level three) is calculated by integrating normative need assessment with impacts on OHRQoL and children's behavioral propensity in terms of appropriate oral hygiene and dental attendance patterns, thus taking into account the effectiveness and appropriateness of suggested treatments in the decision making process. Following this socio-dental approach, Gherunpong et al [10-12] reported that relying on normative methods (i.e. clinical diagnosis) alone without integrating the psychosocial dimensions of oral health, seriously overestimated need for orthodontic treatment in 11–12 year old Thais. Compared to a normative approach to need assessment, the socio-dental approach provided a reduction of 70% in the volume of estimated treatment need [10-12]. Accordingly, a normative measure of orthodontic treatment need estimated by converting clinical measures alone is expected to be too high to be met in a Tanzanian context where the government's oral health care budget is inadequate to meet the increasing oral health needs of the population [21].

**Figure 1:**
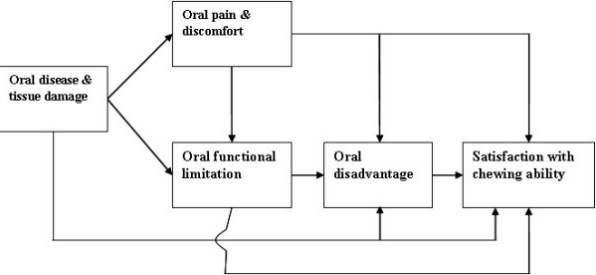
Conceptual model of chewing satisfaction showing associations between oral health constructs (Gilbert et al. 1998).

The present study aims to assess the prevalence and correlates of perceived orthodontic conditions and dissatisfaction with dental appearance/dental function in Tanzanian schoolchildren that are without any history of orthodontic treatment. The conceptual model of Gilbert et al [22] (Fig 1) classifying oral health outcomes into four main levels was applied to organize the independent variables and to guide the analyses. These four levels were as follows; 1) oral disease and tissue damage referring to disorder at the organic level such as active disease or tissue loss, 2) oral pain/discomfort denoting the immediate consequences of disease in terms of physical dysfunction such as the inability to speak, swallow and chew food adequately, 3) oral disadvantage referring to the psychosocial and behavioral consequences of oral diseases, such as difficulties performing daily activities and 4) overall satisfaction with dental health. The final concept of *satisfaction with dental health *is subjects' expressed overall evaluation, incorporating expectations, values and social and cultural background. Following this model, it was hypothesized that reported problems in terms of pain, swallowing, teeth position and spaces of teeth and reported oral impacts on daily performances would increase with increased prevalence of malocclusion. Secondly, it was hypothesized that dissatisfaction with dental appearance/function would increase with increased prevalence of malocclusion, increased frequency of reported problems related to teeth and increased oral impacts on daily performances. Considering that feelings regarding teeth appearance and function are central for need assessment and thus for the planning and implementation of oral health care services in Tanzania, orthodontic treatment need was estimated using a modified integrated socio-dental approach [11].

## Methods

A cross-sectional survey was conducted in Dar Es Salaam, the commercial capital and major sea port of Tanzania, from November 2005 to June 2006. Dar Es Salaam is the most densely populated and socially and culturally heterogenic city in Tanzania. According to the 2002 population and house survey in Tanzania, Dar Es Salaam has a total population of 2.5 million and population density of 1,793 per square km. Dar es Salaam is divided into three districts; Kinondoni, Ilala and Temeke with total population sizes of 1.083,913, 634,924 and 768,451 people respectively. All districts have drinking water with fluoride content of about 1 mg fluoride/L (1 ppm). Kinondoni and Temeke are quite diverse districts in terms of their socio-demographic profile, with the former having higher employment rates, literacy rates and proportions of the population using the most expensive form, electricity, as their main source of energy for cooking [23].

The study population comprised of children attending standard 7 in public primary schools. A stratified proportionate two-stage cluster sampling design with public primary schools as the primary sampling unit was utilized. To obtain a sample of schoolchildren of mixed socio-economic background, schools were selected at random from urban and rural areas in Kinondoni and Temeke districts. Overall, 43 rural- (N = 4,809 standard 7 pupils) and 78 urban primary schools (N = 14.725 standard 7 pupils) were listed in Kinondoni. The corresponding number of schools in Temeke were 22 rural (N = 1707 standard 7 pupils) and 77 urban (N = 14103 standard 7 pupils) schools. A sample size of 1200 school children aged 12–14 yr was calculated to be satisfactory for two sided tests, assuming the prevalence of oral impacts to be 0.40 and 0.50 in children with and without orthodontic anomaly, a significance level of 5%, power of 90% and a design factor of 2 [24]. At the first stage, 4 rural (4/43 n = 755 standard 7 pupils) and 6 urban (6/78, n = 1157 standard 7 pupils) schools in Kinondoni and 1 rural (1/22 n = 184 standard 7 pupils) and 5 urban (5/77, n = 949 standard 7 pupils) schools in Temeke were selected by systematic random sampling using a unified sampling fraction. From a total of 3045 standard 7 pupils available in the selected schools, about 100 students in each selected school (i.e. 1601 students constituting 52.6% of all standard 7 students in the selected schools) and fulfilling the inclusion criteria of being in the defined age range of 12–14 yrs were randomly selected from the accessible classes. Only consenting subjects were included in the study and none of the students invited for participation were ill, had a history of psychiatric problems or were disabled. Ethical clearance was obtained from all relevant persons, authorities and committees in Tanzania. These included written permission and clearance for the study from the Research and Publication Committee of the Muhimbili University College of Health Sciences (MUCHS). Permission to work with school children was obtained from Kinondoni and Temeke municipalities, their respective educational authorities, schools administrations, parents and children.

A structured interview schedule was constructed in English and translated into Swahili by two trained research assistants. Oral health professionals reviewed the interview schedule for semantic, experiential and conceptual equivalence. Sensitivity to culture and selection of appropriate words were considered. The interview schedule was piloted before administration [9]. The model of Gilbert and coworkers [22] linking oral diseases with their functional and behavioral outcomes was applied to identify which factors to consider as determinants of dissatisfaction with dental appearance/function, and to help structure the multivariate regression analysis. The interview schedule in Swahili contained three of the four key concepts derived from this model. Oral pain and discomfort, the second level in Gilbert's model [22], was assessed by asking subjects, whether or not they had experienced problems with pain, tooth position and tooth spaces during the previous 3 months. Response categories were given as (0) no and (1) yes. Problems with swallowing was registered in clinical examination as present = 1 and absent = 0. Oral disadvantage, referring to the third level of Gilbert's model [22], was measured broadly using the eight item Child-OIDP, inventory (e.g. During the previous 3 months – how often have problems with your teeth and mouth caused you any difficulty with; eating, speaking, cleaning teeth, smiling, sleeping, emotional balance, study and social contact). For purpose of cross tabulation and logistic regression analysis the OIDPscore (0–8) was dichotomized as 0/1+, producing the categories (0) "no daily performance affected" and (1) "at least one daily performance affected". The scoring method, reliability and validity of the Kiswahili version of the Child-OIDP inventory have been described in detail in a previous paper [9]. Reported state of teeth was assessed using the categories (1) very good (2) good (3) bad (4) very bad and dichotomized into (0) good (original categories 1,2) and (1) bad (original categories 3,4). Satisfaction with teeth appearance/function was coded on 4-point Likert-scales and recoded further into dummy variables in terms of (0) satisfied and (1) dissatisfied. Overall satisfaction with teeth appearance/functioning was constructed as a sum variable from the 2 variables and dichotomized for use in cross tabulation and logistic regression analysis. Socio-demographics were assessed in terms of place of residence (urban/rural), district (Kinondoni/Temeke), gender, age and parental education. A group variable on parental education was constructed from two dummy variables (0/1) on father's and mother's highest level of education. The independent and dependent variables and the number of subjects according to categories are summarized in Table 1.

**Table 1:** Frequency distribution of independent and dependent variables and their categories in Kinondoni and Temeke districts

Variables	Categories (code)	Kinondoni % (n)	Temeke %(n)	p-value
Sex e	Male (1)	41.1 (412)	36.8 (220)	P = 0.050
	Female (2)	58.9 (591)	63.2 (378)	
Age	12 Yr (1)	26.1 (262)	23.9 (143)	P = 0.033
	13 yr (2)	41.0 (420)	48.5 (290)	
	14 yr (3)	32.0 (321)	27.6 (165)	
Parental education	Both low (1)	38.5 (210)	53.8 (149)	P = 0.000
	One low/one high (2)	24.2 (132)	20.9 (58)	
	Both high (3)	37.2 (203)	25.3 (70)	
Place of residence:	Urban (1)	63.5 (637)	82.3 (492)	P = 0.000
	Rural (2)	36.5 (366)	17.7 (106)	
State of health	Good (0)	93.2 (935)	96.5 (577)	P = 0.003
	Bad (1)	6.8 (68)	3.5 (21)	
Reported problems tooth position	Yes (1)	10.5 (105)	13.9 (83)	P = 0.025
	No (2)	89.5 (898)	86.1 (515)	
Reported problems tooth spaces	Yes (1)	7.8 (78)	7.5 (45)	P = 0.465
	No (2)	92.2 (925)	92.5 (553)	
Problem swallowing	No (0)	93.5 (938)	90.8 (543)	P = 0.030
	Yes (1)	6.5 (65)	9.2 (55)	
Problem pain	No (0)	81.3 (815)	75.9 (454)	P = 0.066
	Yes (1)	18.7 (188)	24.1 (144)	
OHIS debris score	Good = 0	68.0 (682)	61.9 (370)	P = 0.007
	Fair/poor = 1	32.0 (321)	38.1 (228)	
OIDP extent	None = 0	81.5 (817)	54.5 (326)	P = 0.001
	> 1 = 1	18.5 (186)	45.5 (272)	
Malocclusion index (SMO)	No (0)	37.4 (328)	34.0 (163)	P = 0.237
	Yes (1)	62.6 (549)	66.0 (316)	
Dissatisfied appearance/function	Yes (0)	25.6 (257)	19.4 (116)	P = 0.002
	No (1)	74.4 (746)	80.6 (482)	
Reported state of teeth	Good (1)	84.8 (851)	91.6 (548)	P = 0.001
	Bad (2)	15.2 (152)	8.4 (50)	

One trained and calibrated dentist (MM) conducted all clinical examinations in classroom setting with natural daylight as the source of illumination and with an assistant recording the observations. Participants identified with problems that needed treatment were referred or advised to seek treatment at the two municipal hospitals of Kinondoni and Temeke districts and oral health education sessions were provided. Occlusion was registered according to Björk et al., [25], with some modifications by Al-Emran et al., [26]. Caries experience was assessed in accordance with the criteria by the World health Organization [27]. Oral hygiene was assessed using the simplified-Oral Hygiene Index (OHI-S) [28].

Sagittal molar occlusion: the basic Angle classification was used. The intermaxillary relationship of first permanent molars was registered as CL I (normal/neutral) when the mesiobuccal cusp of the maxillary first permanent molar occluded in line with mesiobuccal groove of the mandibular first permanent molar. CL II (distal) or CL III (mesial) molar occlusion was recorded when there was deviation of at least one half cusp width distally or mesially to CL I, respectively. It was recorded as Class I (CL I = 1), II (CL II = 2) and III (CL III = 3), and dichotomized into 0 (CL I) and 1(CL II and III) for use in cross tabulation and logistic regression analysis. When first permanent molars were missing, the registration was considered not applicable. Overjet: the distance from the most labial point of the incisal edge of maxillary right central incisor to the most labial surface of the corresponding mandibular incisor. Positive value (maxillary overjet) was recorded if the upper incisor was ahead of the lower incisor, and negative value (mandibular overjet), was registered if the upper incisor was behind the lower incisor. Maxillary overjet was categorized as 1; 1–4.9 mm (grade 1), 2; 5–8.9 mm (grade 2) and 3: ≥ 9 mm (grade 3). It was considered increased when the value exceeded 5 mm, and dichotomized into 0 < 5 mm and 1 ≥ 5 mm for use in cross tabulation and logistic regression analyses. Mandibular overjet was coded as 0; absent, 1: < 0 to -1.9 mm (grade 1) and 2; ≤ – 2 mm (grade 2) and recoded into 0 = absent and 1 = present (1 and 2). Overbite: the vertical overlap of incisors, measured to the nearest half millimetre vertically from the incisal edge of the maxillary right central incisor to the incisal edge of the corresponding mandibular right incisor. If the right central incisor was missing or fractured, it was substituted by left central incisor. It was coded as 1; 0.1–2.9 mm (grade 1), 2; 3–4.9 mm (grade 2) and 3; > 5 mm (grade 3), then recoded into 0 = absent (< 5 mm) and 1 = present (> 5 mm). It was considered deep bite when the value exceeded 5 mm. Open bite: frontal open bite was recorded when there was no vertical overlap of the incisors, measures to nearest half millimetre. A visible space between antagonistic fully erupted canines, premolars or molars was registered as a lateral open bite. Open bite was coded as 0; absent, 1; 0–1.9 mm (frontal open bite grade 1), 2; ≥ 2 mm (frontal open bite grade 2) and 3; lateral open bite, and recoded into 0 = absent and 1 = present (1, 2 and 3). Lateral crossbite: was registered when one or more buccal cusps of the mandibular canines, premolars and/or molars occluded bucally to the buccal cusps of the maxillary antagonists, recorded either as 1; absent, 2; present unilaterally or 3; present bilaterally. It was then dichotomized into 0 = absent (1) and 1 = present (2 and 3). Scissors bite: registered when any of the maxillary premolars and/or molars totally occluded to the buccal surface of the opposing mandibular teeth. It was recorded as 1 = absent, 2 = present unilaterally or 3 = present bilaterally. It was then dichotomized into 0 = absent (1) and 1 = present (2 and 3). Midline shift: was defined as non-coincident upper and lower midlines when the posterior teeth were in maximum intercuspal relationship. It was coded as (1) absent (2) present when the displacement was at least 2 mm or more and recoded into 0 = absent (1) and 1 = present (2). Crowding: was recorded when the total sum of crowding in the segment was at least 2 mm. It was coded as 1 = absent, 2 = present upper jaw, 3 = present lower jaw and 4 = present both jaws. It was recoded into 0 = absent (1) and 1 = present (2, 3 and 4). Spacing: was recorded when the total spacing was least 2 mm in a segment. It was coded as 1; absent, 2; present upper jaw, 3; present lower jaw and 4; present both jaws. Then it was recoded into 0 = absent (1) and 1 = present (2, 3 and 4).

A sum score of malocclusions (SMO) was constructed for use in logistic regression, based on the diagnosis of the absence (0)/presence (1) of the following recordings; maxillary overjet, mandibular overjet, Class II and Class III molar occlusion, open bite, deep bite, lateral cross bite, midline shift, scissors bite, crowding and spacing.

### Statistical analyses

Data were analyzed using SPSS version 14.0. Test-retest reliability for the clinical parameters and the questionnaire variables was assessed using Cohen's weighted kappa statistics with an independent sample of 71 12–14-year-olds and a time interval of 3 weeks. Internal consistency reliability was assessed in the main sample using Cronbach's alpha. Cross-tabulation, Chi-square statistics, Mc Nemar's statistics and multiple logistic regression analyses were used for bivariate- and multivariate analyses, respectively. To adjust for the effect of the cluster design, data were reanalysed using STATA 9.0 with survey command. P-value for statistical significance was set at 0.05.

## Results

### Sample profile

A total of 1003 children from Kinondoni (63.5% urban, 58.9% girls, mean age 13.1 yr) and 598 children from Temeke (82.3% urban, 63.2% girls, mean age 13.0 yr) completed an extensive personal interview and underwent a full mouth clinical examination. The mean OHI-S scores were 1.0 (sd = 0.53, range 0.0–3.3) in Kinondoni and 1.2 (sd = 0.54, range 0.0–4.2) in Temeke. Table 1 provides the percentage distribution of participants' independent and dependent variables in the districts of Kinondoni and Temeke.

### Reproducibility

Duplicate clinical examinations gave Kappa statistics of 0.74, 0.78, 0.79, 0.82, 0.93 and 0.97 for the OHI-S-, midline shift-, deep bite-, mandibular overjet-, maxillary overjet and spacing scores, respectively. Regarding the scores for open bite, Angle classification, cross bite, scissor bite and crowding, the kappa statistics were 1. Test retest reliability for the 8 Child- OIDP items were in the range 0.7 (emotional state) to 1.00 (eating, speaking, cleaning teeth, sleeping, smiling and social contact). Kappa values for the items assessing satisfaction with teeth appearance and teeth function and self-reported problems with teeth were all 1.00. These figures indicate very good intra-examiner reliability according to Landis & Koch [29].

### Prevalence and correlates of self- reported problems with teeth

The prevalence of malocclusions varied from 22.5% (midline shift) to 0.9% (deep bite). Prevalence of mandibular overjet and crowding were statistically significantly higher in children who were dissatisfied with dental appearance and function than in their counterparts who were satisfied. A total of 63.8% had at least one type of anomaly (i.e. scored above zero on the SMO score) and the prevalence was higher in dissatisfied- than in satisfied children (71.6% versus 62.5%, p < 0.001). Moderate proportions of the children investigated confirmed problems with pain (20.7%), teeth position (11.7%) and problems with spaces (7.7%). Moreover, a total of 7.5% of the children were observed with swallowing problems, whereas 28.6% had at least one oral impact (OIDP > 0) (not in table). After controlling for possible confounding effects of socio-demographic factors, the odds ratios for confirming problems with teeth position, spaces, pain and swallowing were respectively 6.7, 3.9 and 1.4, and 6.8 if having any occlusion anomaly (SMO > 0) compared to being without such anomaly (Table 2, 3). Problems related to teeth position were consistently more frequently reported among children in Temeke than among their counterparts in Kinondoni (Table 2, 3).

**Table 2:** Percentage and OR (95% CI) of participants who reported problem with position- and spaces of teeth by socio demographic variables and malocclusion index, SMO.

	Tooth position % (n)	Adjusted step OR (95% CI	Space % (n)	Adjusted step OR (95% CI)
*Socio demographics*				
Kinondoni	10.5 (105)	1	7.8 (78)	1
Temeke	13.9 (83)*	1.6 (1.0–2.5)	7.5 (45)	0.9 (0.5–1.6)
Boy	13.1 (83)	1	8.4 (53)	1
Girl	10.8 (105)	0.7 (0.4–1.0)	7.2 (70)	1.1 (0.6–1.7)
Urban	11.3 (128)	1	8.4 (95)	1
Rural	12.7 (60)	1.1 (0.7–1.8)	5.9 (28)	0.7 (0.4–1.3)
12 yr	11.9 (48)	1	7.7 (31)	1
13 yr	12.5 (89)	0.8 (0.4–1.4)	6.8 (48)	0.7 (0.3–1.3)
14	10.5 (51)	0.7 (0.3–1.3)	9.1 (44)	1.1 (0.5–1.9)
Both parents low education	14.2 (51)	1	8.9 (32)	1
One low/one high	8.9 (17)	0.5 (0.2–0.9)	11.6 (22)	1.3 (0.7–2.3)
Both parents high education	13.2 (369	0.8 (0.5–1.3)	8.8 (24)	0.9 (0.5–1.7)
*Clinical status*				
SMO = 0 (no malocclusion diagnosed)	1.6 (8)	1	2.9 (14)	1
SMO > 0	16.4 (142)**	6.7 (3.3–13.3)	8.8 (76)**	3.9 (2.0–7.8)

**p < 0.001, *p < 0.05

**Table 3:** Percentage and OR (95% CI) of participants who reported problem with swallowing and pain by socio demographic variables and malocclusion index, SMO.

Variables	Problem with swallowing % (n)	Adjusted step OR (95% CI	Problem with pain % (n)	Adjusted step OR (95% CI)
*Socio demographics*				
Kinondoni	6.5 (65)	1	18.7 (188)	1
Temeke	9.2 (55)*	1.3 (0.7–2.3)	24.1 (144)**	1.5 (1.1–2.1)
Boy	6.6 (42)	1	19.3 (12)	1
Girl	8.0 (78)	1.5 (0.8–2.7)	21.7 (210)	1.3 (0.9–1.8)
Urban	8.4 (95)	1	20.9 (236)	|
Rural	5.3 (25)*	0.4 (0.2–0.8)	20.3 (96)	1.2 (0.8–1.8)
12 yr	7.2 (29)	1	18.0 (73)	1
13 yr	6.1 (43)	0.8 (0.4–1.7)	20.1 (143)	1.2 (0.7–1.8)
14	9.9 (48)	1.1 (0.5–2.3)	23.9 (116)	1.3 (0.8–2.1)
Both parents low education	8.4 (30)	1	20.3 (73)	1
One low/one high	7.4 (14)	0.9 (0.4–1.8)	21.6 (41)	1.1 (0.7–1.7)
Both parents high education	7.0 (19)	0.8 (0.4–1.6)	20.5 (56)	1.1 (0.7–1.7)
*Clinical status*				
SMO = 0 (at least one malocclusion diagnosed)	1.0 (5)	1	17.1 (87)	1
SMO > 0	5.3 (46)**	6.8 (2.7–17.4)	21.0 (182)**	1.4 (1.0–2.0)

**p < 0.001, *p < 0.05

### Prevalence and correlates of dissatisfaction with dental appearance/functioning

In total, 23.3% (373/1601) children were dissatisfied with their dental appearance/function. The corresponding figures in Kinondoni and Temeke were 25.6 (257/1003) and 19.4 (116/598), respectively. Table 4 depicts unadjusted and adjusted OR from binary and multiple logistic regression analysis of children being dissatisfied with their dental appearance/function according to socio-demographic-, clinically assessed malocclusion, reported dental problems and oral disadvantage variables. Age, gender, district, place of residence and parental education were entered into step one providing a Nagelkerke's R^2 ^of 0.016 (Model Chi square: 9.133, df = 7, p = 0.243). Entering the SMO index in step two raised the Nagelkerke's R^2 ^to 0.026 (Model Chi square: 14,546, df = 8, p = 0.069). Entering four variables of reported dental problems in step three and the OIDP score and self rated health in step four raised the Nagelkerke's R^2 ^to 0.095 (Model Chi square 54.926, df = 12, p < 0.001) and to 0.139 (Model Chi square = 81.379, df = 14, p < 0.001), respectively. In the final model, dissatisfied children were less likely to be from Temeke (OR = 0.5), having both parents with high education (OR = 0.6), reporting problems with teeth positioning (OR = 4.3), having at least one oral impact (OR = 2.2) and confirming bad health status (OR = 2.7). Although the SMO index discriminated statistically significantly between satisfied and dissatisfied children at the bivariate level, the clinical variable did not maintain its statistically significant effect in the final regression model.

**Table 4:** Unadjusted and adjusted odds ratio (OR) and 95% confidence interval (CI) of being dissatisfied with dental appearance and function according to socio-demographics (step 1), clinically assessed criteria of malocclusion, SMO (step 11) and subject-rated oral health (step III)

Variables	Unadjusted OR (95% CI)	Adjusted step I OR (95% CI
*Step I (socio demographics R*^2^=		
Kinondoni	1	1
Temeke	0.7 (0.5–0.8)	0.5 (0.3–0.8)
Boy	1	1
Girl	0.9 (0.7–1.1)	1.0 (0.7–1.4)
Urban	1	1
Rural	0.9 (0.7–1.2)	0.7 (0.4–1.0)
12 yr	1	1
13 yr	1.0 (0.8–1.4)	0.8 (0.5–1.3)
14	1.2 (0.8–1.6)	0.9 (0.7–1.4)
Both parents low education	1	1
One low/one high	0.7 (0.4–1.1)	0.7 (0.4–1.1)
Both parents high education	0.6 (0.4–0.9)	0.6 (0.4–0.9)
*Step II (clinical status)*		
SMO = 0	1	1
SMO > 0	1.6 (1.2–1.9)	1.2 (0.6–1.6)
*Step III (reported problems)*		
Pain : no	1	1
Pain: yes	1.4 (1.1–1.8)	0.8 (0.5–1.3)
Problem swallowing: no	1	1
Problem swallowing: yes	1.5 (1.0–2.1)	1.1 (0.5–1.9)
Problem position: no	1	1
Problem position: yes	3.4 (2.5–4.7)	4.3 (2.7–6.9)
Problem spaces: no	1	1
Problem spaces: yes	1.7 (1.1–2.4)	1.4 (0.8–2.3)
*Step IV (oral disadvantage)*		
OIDP = 0	1	1
OIDO > 0	1.8 (1.4–2.3)	2.2 (1.4–3.1)
Self rated health: good	1	1
Self rated health: bad	3.3 (2.1–5.1)	2.7 (1.5–5.1)

### Normative-, impact-, and propensity related need for orthodontic treatment

A total of 63.8 % (865/1601) children fulfilled the criteria for professionally judged normative treatment need in terms of having at least one diagnosed malocclusion (i.e. SMO > 0). In turn, a total of 18.9% (303/1601) children fulfilled the criteria of impact related treatment need, i.e. having normative treatment need and also reporting impacts on daily performances related to malocclusion. Finally, a total of 12% (8.4% in Kinondon and 18.1% in Temeke) (192/1601) had propensity related need, i.e. having impact related need and good behavioral propensity in terms of satisfactory oral hygiene scores. Thus, they should be treated as initially planned. For those children who fulfilled the criteria for impact related need but did not have high propensity (6.9% or 111/1601), oral health promotion should be offered and orthodontic treatment delayed until their oral hygiene improves in terms of maintenance of adequate oral hygiene scores. Mc Nemar test revealed statistically significant differences between the normative need estimate on the one hand side and the impact- and propensity related need estimates on the other (p < 0.001).

## Discussion

This is one of the first studies to systematically investigate the psycho-social impacts of malocclusion and orthodontic socio-dental needs among children in a sub-Saharan African country. A comparison of the sex-and parental education characteristics of the Kinondoni and Temeke study participants with the corresponding data for the target populations indicated that the study sample was broadly representative of the populations of school going children 12–14 yr in those districts. In spite that the prevalence of overall malocclusion was relatively high (63.8%), only a minority reported dissatisfaction with dental appearance/function (23.3%), confirmed dental problems- (7.5%–21%) and had oral impacts on daily performances (overall prevalence 29%) (not in table). As shown in Table 2 and 3, all self-reported dental problems were positively and statistically significantly associated with the measure of normative orthodontic treatment need after controlling for socio-demographic factors (p < 0.001). Thus, the risk of reporting problems if having any malocclusion (SMO > 0) varied from OR 1.4 with respect to dental pain to OR = 6.8 regarding problems with swallowing (Table 3). In accordance with previous studies considering the psycho-social impacts of children's orthodontic status, the present results suggest that malocclusion associates with perceived orthodontic status, dental symptoms and the oral health domain of appearance/functional limitations in Tanzanian children [29-31]. Whereas malocclusion does not cause dental pain directly, it has been suggested that it gives rise to pain indirectly by causing temporo-mandibular disorder (TMD) and dental-, gingival- and mucosal trauma [4]. In the present study, malocclusion was related to swallowing problems. Such problems might affect food choices and finally deteriorate children's nutritional status. A review of eight studies revealed that, malocclusion was positively associated with diet and malnutrition [32].

Notably, large proportions of children with a normative treatment need did not confirm any psycho-social impact. For example 83% and 94% of children with SMO > 0 did not report problems with teeth positions and swallowing, respectively. This supports previous studies showing that children and adolescents are less concerned with their malocclusion than professionals and have lower threshold to detect malocclusion traits [33]. In evaluating a questionnaire to measure oral quality of life in 11–14 year old children, Jokovic et al [5] found the mean child perception questionnaire score (CPQ) to be comparably low in children with malocclusion. Most studies have shown that using clinical criteria for the estimation of diagnosis of malocclusion overestimates the problem when compared with individuals' perception [10-12]. However, the results of a Brazilian study focusing 10 to 14-year-olds came to a different conclusion, in that 87% of the children perceived a need for orthodontic treatment, whereas the normative treatment need was only 52% [13]. Whilst there may be less direct impact on quality of life indicators from malocclusion among children, by early adulthood young people will probably think differently about the impact on their dental appearance and function.

Malocclusion when used in combination with perceived dental problems and other psycho-social impact scores explained significantly more of children's concern about their dental appearance/function than did the clinical measure of occlusal status alone. The results from multivariate logistic regression analysis support Gilbert's [22] model in that dissatisfaction scores were influenced, statistically significantly but differently by at least one variable from each oral health outcome domain. Reported problem with teeth position was the strongest predictor (OR = 4.3), followed in descending order by self-rated health status (OR = 2.7) and OIDP scores (OR = 2.2) (Table 4). In the bivariate model, but not in the final multiple regression analysis, children with SMO > 0 had a higher probability than their counterparts without to be dissatisfied with dental appearance/function. Consistent with previous studies, the present one indicated that crowding and mandibular overjet were the conditions of most concern to Tanzanian children [15,18,29-34]. As shown in Table 2 and 3, the overall malocclusion scores showed positive associations with perceived orthodontic status and symptoms after controlling for socio-demographic factors, indicating that in the final analysis (Table 4) these variables have mediated the effect of malocclusion upon dissatisfaction scores. Temeke children were more likely than their Kinondoni counterparts to confirm problems with teeth position, with pain and swallowing and to report oral impacts. Nevertheless, less affluent children from Temeke and children having parents with higher education were, irrespective of diagnosed malocclusion and its psycho-social impacts, less likely than their counterparts in the opposite groups to confirm dissatisfaction. This suggests that children's concern about their dental appearance is influenced by the social and cultural context in which they live. It is evident for instance that spacing is disliked in white cultures but considered a sign of beauty in many African cultures [15,18]. Locker reported on socio-economic disparities in children's oral quality of life, with children from low income households having the poorest oral health related quality of life [35].

Children's feelings concerning their dental appearance and function corresponds to broader concepts of oral health and are thus central to the assessment of orthodontic treatment need [3]. Consistent with what has been reported among Tanzanian adults with respect to needs for prosthodontic treatment, children's estimated normative orthodontic treatment need decreased markedly when a socio-dental approach was used [36]. Among the children with a normative need defined as any type of dental irregularity, SMO > 0, (63.8%), only 18.9% had an impact related need and 12% had high propensity related need, indicating that about one fifth of those with any malocclusion would actually demand some kind of orthodontic care. A minority of the children had low propensity (6.9%) and should initially be offered an alternative intervention with oral health education. The estimated normative need by far exceeded a more realistic estimate based on a modified version of an integrative socio-dental approach. This result corroborates those of a previous study using the same socio-dental approach to estimate orthodontic treatment need among Thai children in that normative need for orthodontic care was found to be much higher (35%) than the medium to high propensity related need (18.9%) [10-12]. High amounts of children's normative treatment need have also been reported from other countries, ranging from 38% among primary school children in Turkey to 57% and 30% in respectively 9 year old- and 12 year old children from UK, for review see [37,38]. Caution should be made when comparing the estimates of normative need made in this study with those in other studies using the IOTN index for need assessment. Some caution should also be taken when evaluating the results from the present study since the overall- and not a malocclusion specific OIDP score attributing oral impacts to malocclusion, was used in the analyses. However, the overall oral disease burden among the Tanzanian children investigated was not high [9]. Thus, it is less likely that other oral conditions commonly found in children have contributed much into the overall OIDP scores. Finally only one of two behaviors (i.e. oral hygiene but not dental attendance) was utilized to assess propensity related need. As there is no dental health care service offered on a regular basis to children in Tanzania, behaviors related to compliance with dental appointments were not considered appropriate for use in the present study.

## Conclusion

In conclusion, contrary to the prevalence of malocclusion, reported psycho social impacts and dissatisfaction with appearance/function was not very frequent among Tanzanian primary schoolchildren. Subjects with malocclusion reported problems most frequently and malocclusion together with other psycho-social impact scores determined children's overall evaluation of their dental appearance and function. Finally, a marked difference was found between the standard normative- and socio-dental need assessment approaches with socio-dental needs being five times lower than the standard normative need assessment.

## Competing interests

The authors declare that they have no competing interests.

## Authors' contributions

ANÅ conceived of the study and drafted the manuscript. MM carried out the data collection, made the data amenable for statistical analyses, analyzed the data and have contributed to the development of the paper. She has critically revised the intellectual content of the manuscript. PB has been involved in drafting the manuscript and has critically revised its intellectual content.

## Pre-publication history

The pre-publication history for this paper can be accessed here:


